# High myoglobin level as an independent risk factor for death in elderly patients with critical COVID-19 infection: a retrospective case-control study

**DOI:** 10.1186/s12879-024-09621-5

**Published:** 2024-08-20

**Authors:** Xiaoxiao Sun, Hui Zhang, Meixian Zhang, Miaomiao Fei, Lize Xiong, Cheng Li

**Affiliations:** 1 Department of Critical Care Medicine, Shanghai Key Laboratory of Anesthesiology and Brain Functional Modulation, Clinical Research Center for Anesthesiology and Perioperative Medicine, Translational Research Institute of Brain and Brain-Like Intelligence, Shanghai Fourth People’s Hospital, School of Medicine, Tongji University, No. 1279, Sanmen Road, Hongkou District, Shanghai, 200434 China; 2 Department of Anesthesiology and Perioperative Medicine, Shanghai Key Laboratory of Anesthesiology and Brain Functional Modulation, Clinical Research Center for Anesthesiology and Perioperative Medicine, Translational Research Institute of Brain and Brain-Like Intelligence, Shanghai Fourth People’s Hospital, School of Medicine, Tongji University, Shanghai, 200434 China

## Abstract

**Aim:**

This study aimed to discover risk factors for death in patients with critical COVID-19 infection in order to identify patients with a higher risk of death at an early stage.

**Methods:**

We retrospectively analyzed the clinical data of patients with critical COVID-19 infection from April 2022 to June 2022. Data were collected from the electronic medical records. Propensity matching scores were used to reduce the effect of confounding factors, such as patient baseline variables. Independent risk factors affecting patient prognosis were assessed using univariate logistic regression and multivariate logistic regression analysis. Restricted cubic spline curves were used to assess the relationship between independent and dependent variables.

**Results:**

The data of 132 patients with critical COVID-19 infection were included in the study. Of the 132 patients, 79 survived and 53 died. Among laboratory indicators, patients who died had higher proportions of abnormalities in procalcitonin, aspartate aminotransferase (AST), creatinine, cardiac troponin I, and myoglobin. Univariate and multivariate logistic regression analyses suggested that abnormal AST (OR = 4.98, *P* = 0.02), creatinine (OR = 7.93, *P* = 0.021), and myoglobin (OR = 103.08, *P* = 0.002) were independent risk factors for death. After correction for AST and creatinine, a linear relationship between myoglobin and risk of death in patients was found using restricted cubic splines.

**Conclusion:**

High myoglobin level is an independent risk factor for death and is therefore a prognostic marker in elderly patients with severe COVID-19 infection.

## Introduction

In the spring of 2022, the city of Shanghai experienced an outbreak of coronavirus disease (COVID-19), with the prevalent variant of the severe acute respiratory syndrome coronavirus 2 being the Omicron BA.2 (Omicron BA.2) strain. Recently, the COVID-19 has appeared frequently even if there was possibly mutation. Patients with COVID-19 have a wide range of clinical presentations [1], with 81% being mild; 14%, severe; and 5%, critical (respiratory failure, infectious shock, and/or multi-organ dysfunction) infections. A study of 20,133 patients hospitalized for COVID-19 infection in the United Kingdom [2] reported that 17.1% were admitted to the intensive care unit (ICU). According to the data, the overall in-hospital mortality rate for COVID-19 was approximately 15–20%. However, it was as high as 40% in patients with infections requiring ICU admission and more than 89% in patients aged 60–80 years hospitalized with infections [3].

Previous findings suggest that [4] elevated D-dimer, CK-MB, cardiac troponin I (cTnI), and C-reactive protein (CRP) levels increase the risk of death in patients with COVID-19 pneumonia. Abnormal D-dimer, cTnI and CRP levels in patients on admission, glomerular filtration rate, lactate dehydrogenase, CRP, neutrophil count, lymphocyte count, and monocyte count are also confounding factors. The risk of death in critically ill patients with COVID-19 infection can be predicted to some extent [5–8]. Several studies have focused on risk factors for disease severity or risk factors for death in patients with COVID-19 infection. Because patients with critical COVID-19 infection have a high mortality rate, it is important to stratify the risk of death in this population to allow the early identification of patients with a higher risk of death in order to ensure different patients can receive different medical treatments and medical resources can be allocated more rationally. This study aimed to analyze the clinical characteristics of patients with critical COVID-19 infection who died or survived, as well as investigate the risk factors for death in order to identifying the death risk of patients.

## Patients and methods

### Patients

This study retrospectively analyzed the clinical data of patients with the critical type of COVID-19 infection who were hospitalized in the ICU of Shanghai Fourth People’s Hospital, Tongji University, from April 2022 to June 2022. The study was reviewed and approved by the Biomedical Ethics Committee of the Fourth People’s Hospital of Shanghai (Review and Approval No: SYLL2023082). Participant data were collected from the electronic medical record system, including (1) general data: sex, age; (2) clinical admission data: underlying disease, referral, vaccination status, days of hospitalization, prognosis; (3) laboratory markers within 48 h of ICU admission: routine blood count, liver and kidney function, myocardial markers, coagulation function, inflammatory indices, inflammatory factors, etc.

Patients were recruited in the analysis based on the following inclusion and exclusion criteria. The inclusion criteria comprised meeting the diagnostic criteria of the “Diagnostic Protocol for Novel Coronavirus Pneumonia (Trial 10th Edition)” issued by the National Health and Wellness Commission and admitted to the ICU. Moreover, those who met any of the following diagnostic criteria were considered to have critical COVID-19 infection: (1) respiratory failure requiring mechanical ventilation, (2) shock, (3) organ failure requiring intensive care monitoring, and (4) age ≥ 18 years. The exclusion criteria included (1) family members who voluntarily signed a consent form to withhold active resuscitation or other serious underlying diseases that led to death; (2) pregnant women or patients during pregnancy; (3) non-critical COVID-19 infection; and (4) ineligibility for inclusion in the study for various reasons in the opinion of the investigator.

### Statistical procedures

Normally distributed continuous variables are expressed as mean ± standard deviation and were analyzed using Student’s t-test. Non-normally distributed continuous variables are expressed as median (interquartile range) and were analyzed using the Mann–Whitney U test. Categorical variables are expressed as percentages and were analyzed with the χ2 test. Propensity score matching was used to reduce the effect of confounding factors, such as patient baseline variables. Independent risk factors affecting patient prognosis were assessed using univariate and multivariate logistic regression. Restricted cubic spline curves were used to assess the relationship between independent and dependent variables. *P* < 0.05 was considered a significant difference. Data analysis was performed in R 4.2.2 (R Foundation for Statistical Computing, Vienna, Austria). Propensity matching scores were analyzed using the MatchIt package and logistic regression analysis. Restricted cubic spline curves were performed using the rms package.

## Results

Data were collected from 137 critically ill COVID-19 patients, of which 84 survived and 53 died. However, the majority of patients were over the age of 60. In order to reduce the effect of age on the prognosis of the patients, we excluded cases with an age of less than 60 years. Consequently, 132 patients were ultimately included in the study, of which 79 survived and 53 died. To ensure the test indicators were within the normal reference range, the reference values were divided into normal and abnormal values. The baseline information is shown in Table 1, and the laboratory test parameters are presented in Table 2. In the baseline data, the number of cases of previous hypertension (*P* = 0.001), diabetes mellitus (*P* = 0.019), and stroke (*P* = 0.038) was lower in patients in the death group than in the survivor group. Among laboratory parameters, patients in the death group had more abnormalities in procalcitonin (dead vs. alive: 60.4%/34.2%, *P* = 0.005), AST (dead vs. alive: 69.8%/25.3%, *P* < 0.001), creatinine (dead vs. alive: 86.8%/64.6%, *P* = 0.008), potassium (dead vs. alive: 34%/6.3%, *P* < 0.001), and cTnI (dead vs. alive: 84. 9%/59.5%, *P* = 0.003). In addition, myoglobin (dead vs. alive: 94.3%/67.1%, *P* < 0.001), CK-MB (dead vs. alive: 52.8%/29.1%, *P* = 0.01), and PT (dead vs. alive: 54.7%/35.4%, *P* = 0.044) levels were proportionally more abnormal with significant statistical differences (*P* < 0.05) in the death group than in the surviving group.

**Table 1:** The baseline characteristics of collected patients admitted to ICU

Variables	Alive (*n* = 79)	Dead (*n* = 53)	*P* value	Variables	Alive (*n* = 79)	Dead (*n* = 53)	*P* value
**Age(years)**	86[75.0, 90.0]	87.0[76.0,91.0]	0.517	**Coronary Disease**			0.243
**Gender**			0.615	Yes	33 (41.8)	16 (30.2)	
Male	45 (57.0)	27 (50.9)		No	46(58.2)	37 (69.8)	
Female	34 (43.0)	26 (49.1)		**Mental diseases**			1.000
**Hypertension**			0.001*	Yes	5(6.3)	3 (5.7)	
Yes	64 (81)	27 (50.9)		No	74(93.7)	50 (94.3)	
No	15 (22.6)	26 (49.1)		**Vaccination (Dose)**			0.663
**Diabetes**			0.019*	0	74 (93.7)	50 (94.3)	
Yes	22 (27.8)	5 (9.4)		1	1 (1.3)	0 (0.0)	
No	57 (72.2)	48 (90.6)		2	3 (3.8)	3 (5.7)	
**Apoplexy**			0.038*	3	1 (1.3)	0 (0.0)	
Yes	41(51.9)	17 (32.1)		**Ventilator**			0.074
No	38(48.1)	36 (67.9)		Yes	16(20.3)	19 (35.8)	
				No	63(79.7)	34 (64.2)	

(* *P* < 0.05)

**Table 2:** The laboratory biomarkers of collected patients admitted to ICU

Variables	Alive (*n* = 79)	Dead (*n* = 53)	*P* value	Variables	Alive (*n* = 79)	Dead (*n* = 53)	*P* value
WBC(*10^9/L)			0.470	**Creatinine(umol/L)**			0.007*
3.5–9.5	33(41.8)	18 (44.0)		57–97	28(35.4)	7 (13.2)	
< 3.5/>9.5	46(58.2)	35 (66.0)		< 57 / >97	51(64.6)	46 (86.8)	
RBC(*10^12/L)			0.337	**GFR(ml/min/1.73m** ^**2**^ **)**			0.055
4.3–5.8	16 (20.3)	6 (11.3)		90–120	16 (20.3)	5 (7.5)	
< 4.3/>5.8	63 (79.7)	47 (88.7)		< 90 / >120	63 (79.7)	49 (92.5)	
**Hb(g/L)**			0.464	**Kalium(mmol/L)**			< 0.001*
130–175	11(13.9)	4 (7.5)		3.5–5.5	74 (93.7)	35 (66.0)	
< 130/>175	68 (86.1)	49 (92.5)		< 3.5 / >5.5	5 (6.3)	18 (34.0)	
**RDW SD**			0.269	**Sodium(mmol/L)**			0.062
37–50	56(70.9)	31 (58.5)		135–145	51 (64.6)	26 (49.1)	
< 37/>50	23 (29.1)	22 (41.5)		< 135 / >145	28 (35.4)	27 (50.9)	
PLT(*10^9/L)			0.807	**cTnI(ng/mL)**			0.001*
125–350	56(70.9)	36 (67.9)		< 0.028	32 (40.5)	8 (15.1)	
< 125/>350	23(29.1)	17 (32.1)		> 0.028	47 (59.5)	45 (84.9)	
**CRP(mg/L)**			0.336	**Myoglobin(ug/L)**			0.002*
≤ 6	5 (6.3)	1 (1.9)		20.56–72.48	26 (32.9)	3 (5.7)	
> 6	74(93.7)	52 (98.1)		< 20.56/ >72.48	53 (67.1)	50 (94.3)	
**Procalcitonin(ng/mL)**			0.003*	**CK-MB(U/L)**			0.005*
< 0.5	52(65.8)	21 (39.6)		≤ 4.88	56 (70.9)	25 (47.2)	
≥ 0.5	27(34.2)	32 (60.4)		> 4.88	23 (29.1)	28 (52.8)	
**IL-6(pg/mL)**			1.000	**PT(s)**			0.044*
≤ 6.6	2 (2.5)	1 (1.9)		9.4–12.5	41 (64.6)	25 (45.3)	
> 6.6	77(97.5)	52 (98.1)		< 9.4/ >12.5	38 (35.4)	29 (54.7)	
**ALT(U/L)**			0.379	**TT(s)**			1.000
9–50	63(79.7)	38 (71.7)		13–21	67 (84.8)	47 (86.8)	
< 9 / >50	16(20.3)	15 (28.3)		< 13/ >21	12 (15.2)	7 (13.2)	
**AST(U/L)**			< 0.001	**APTT(s)**			1.000
15–40	59(74.7)	17 (30.2)		23.5–40.7	67 (84.8)	44 (83.0)	
< 15 / >40	20 (25.3)	37 (69.8)		< 23.5/ >40.7	12 (15.2)	9 (17.0)	
**r-GTT(U/L)**			0.642	**Fibrinogen(mg/dL)**			1.000
10–60	65(82.3)	41 (77.4)		2–5	58 (73.4)	40 (75.5)	
< 10 / >60	14 (17.7)	12 (22.6)		< 2/ >5	21 (26.6)	13 (24.5)	
**BUN(mmol/L)**			0.132	**D-Dimer(mg/L)**			0.689
3.1-8.0	25 (31.6)	10 (18.9)		≤ 0.5	2 (2.5)	0 (0.0)	
< 3.1 / >8.0	54 (68.4)	43 (81.1)		> 0.5	77 (97.5)	53 (100.0)	
**HR(mean(SD))**	94.66(21.63)	98.87(26.42)	0.318	**SPO**_**2**_ **(mean(SD))**	95.91(5.17)	94.70(7.37)	0.268
**T(mean[IQR])**	36.6[36.2, 36.95]	36.6[36.3, 37.1]	0.485				

(* *P* < 0.05)

**Fig. 1:**
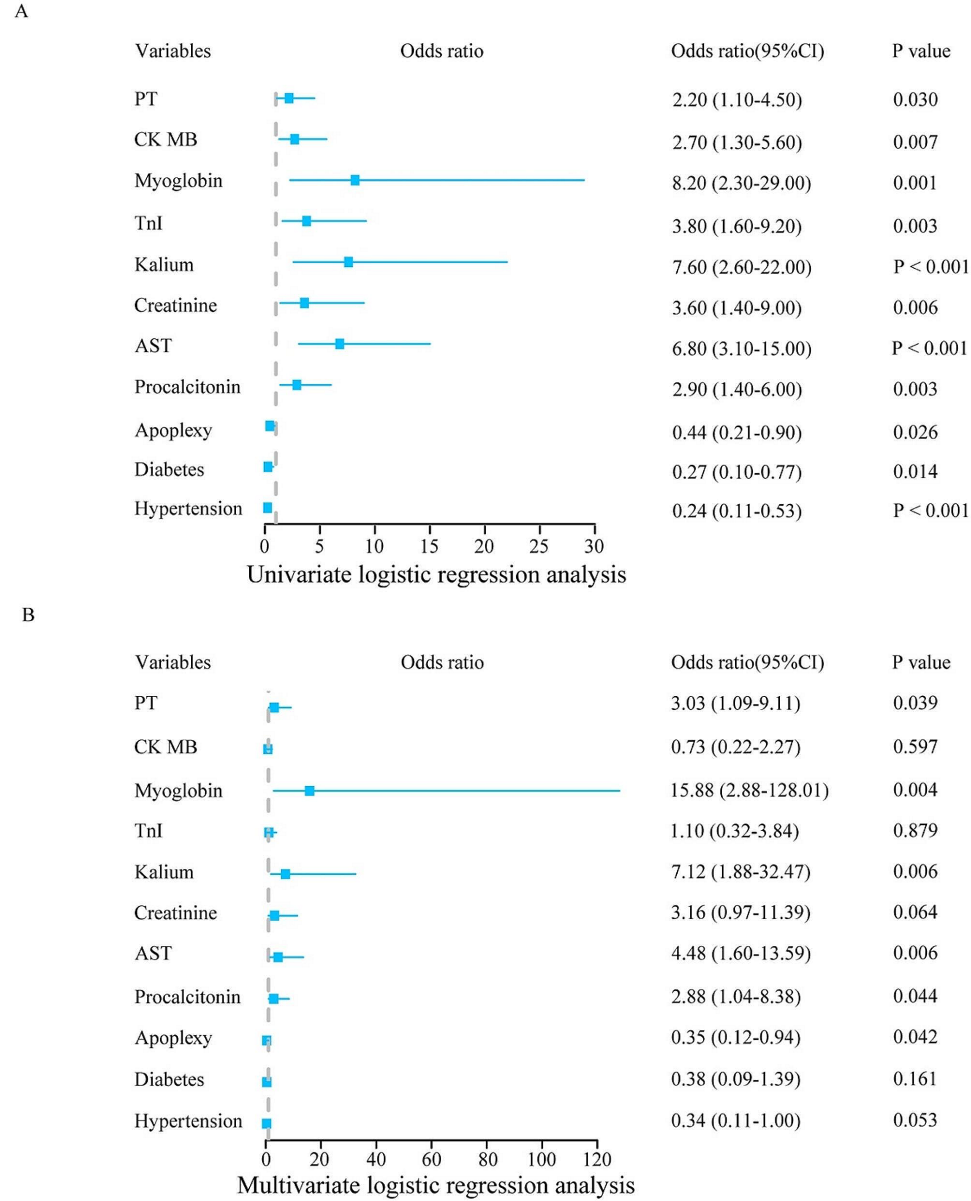
Logistic regression analysis. **A**: The outcome of univariate logistic regression analysis. **B**: The outcome of multivariate logistic regression analysis

To explore the independent factors for outcomes of critically infected patients, univariate and multivariate logistic regression analyses were applied based on the statistical differences variables, such as hypertension, diabetes mellitus, apoplexy, procalcitonin, AST, creatinine, potassium, cTnI, myoglobin, CK-MB, and PT. The results of the univariate logistic regression analysis showed that all selected variables were associated with the outcome of patients. Multivariate logistic regression analysis showed that apoplexy, procalcitonin, AST, potassium, PT and myoglobin were independent risk factors of death in the patients; however, apoplexy was a protective factor (Table 3; Fig. 1). The result showed that a significant selection bias existed during data collection because infected patients with hypertension, diabetes, or apoplexy were prone to poor outcomes.

**Table 3:** Results of univariate and multivariate logistic regression analysis with significantly differential variants between all dead and alive patients

Variants	Univariate logistic regression			multivariate logistic regression	
	OR (95%CI)	*P* value		OR (95%CI)	Adj. *P* value
**Hypertension**	0.24 (0.11–0.53)	0.00037*		0.34 (0.11-1.00)	0.053
**Diabetes**	0.27 (0.095–0.77)	0.014*		0.38 (0.09–1.39)	0.161
**Apoplexy**	0.44 (0.21–0.90)	0.026*		0.35 (0.12–0.94)	0.042*
**Procalcitonin**	2.90 (1.4-6.00)	0.0034*		2.88 (1.04–8.38)	0.044*
**AST**	6.80 (3.10–15.00)	< 0.001*		4.48 (1.60-13.59)	0.006*
**Creatinine**	3.60 (1.40-9.00)	0.0062*		3.16 (0.97–11.39)	0.064
**Kalium**	7.6 (2.60–22.00)	< 0.001*		7.12 (1.88–32.47)	0.006*
**cTnI**	3.80 (1.60–9.20)	0.0027*		1.10 (0.32–3.84)	0.879
**Myoglobin**	8.20 (2.30–29.00)	0.001*		15.88 (2.88-128.01)	0.004*
**CK-MB**	2.70 (1.30–5.60)	0.0067*		0.73 (0.22–2.27)	0.597
**PT**	2.20 (1.10–4.50)	0.03*		3.03 (1.09–9.11)	0.039

(* *P* < 0.05)

Subsequently, we used matching scores with 1:1 to eliminate the effect of the underlying disease and confounding factors, such as hypertension, diabetes, and apoplexy, to further search for independent risk factors for patient prognosis. After eliminating the underlying disease, 80 patients were screened during the analysis, and no significant difference in underlying disease was detected between the two groups (Table 4). The results from laboratory indicators suggested that patients in the death group, compared to their counterparts in the surviving group, had higher proportions of abnormalities in RDW SD (dead vs. alive: 45%/17.5%, *P* = 0.016), AST (dead vs. alive: 70%/20.0%, *P* < 0.001), creatinine (dead vs. alive: 87.5%/62.5%, *P* = 0.020), kalium (dead vs. alive: 35.0%/7.5%, *P* = 0.006), cTnI (dead vs. alive: 87.5%/62.5%, *P* = 0.020), and myoglobin (dead vs. alive: 95.0%/57.5%, *P* < 0.001) (Table 5).

**Table 4:** The baseline characteristics of patients after PSM

Variables	Alive (*n* = 40)	Dead (*n* = 40)	*P* value	Variables	Alive (*n* = 40)	Dead (*n* = 40)	*P* value
**Age(years)**	85.50 [74.50, 89.25]	87.50[76.75, 90.25]	0.353	**Coronary Disease**			1
**Gender**			0.500	Yes	15 (37.5)	15 (37.0)	
Male	24(60.0)	20(50.0)		No	25 (62.5)	25 (62.5)	
Female	16 (40.0)	20 (50.0)		**Mental diseases**			0.065
**Hypertension**			1	Yes	5(7.5)	0 (0)	
Yes	27 (67.5)	27 (67.5)		No	35(92.5)	40(100)	
No	13(32.5)	13(32.5)		**Vaccination(Dose)**			0.504
**Diabetes**			1	0	37(92.5)	38(95.0)	
Yes	5 (12.5)	5 (12.5)		1	1 (2.5)	0 (0.0)	
No	35(87.5)	35(87.5)		2	1 (2.5)	2 (5.0)	
**Apoplexy**			1	3	1 (2.5)	0 (0.0)	
Yes	16 (40.0)	16 (40.0)		**Ventilator**			0.439
No	24 (60.0)	24 (60.0)		Yes	8(20)	12(30)	
				No	32(80)	28(70)	

**Table 5:** The laboratory biomarkers of patients after PSM

Variables	Alive (*n* = 40)	Dead (*n* = 40)	*P* value	Variables	Alive (*n* = 40)	Dead (*n* = 40)	*P* value
WBC(*10^9/L)			0.819	**Creatinine(umol/L)**			0.020*
3.5–9.5	17 (42.5)	15 (57.5)		57–97	15 (37.5)	5 (12.5)	
< 3.5/>9.5	23(57.5)	25(62.5)		< 57 / >97	25 (62.5)	35 (87.5)	
RBC(*10^12/L)			0.377	**GFR(ml/min/1.73m** ^**2**^ **)**			0.069
4.3–5.8	9 (22.5)	5 (12.5)		90–120	10 (25.0)	3 (7.5)	
< 4.3/>5.8	31 (77.2)	35 (87.5)		< 90 / >120	30 (75.0)	37 (92.5)	
**Hb(g/L)**			0.709	**Kalium(mmol/L)**			0.006
130–175	5 (12.5)	3 (7.5)		3.5–5.5	37 (92.5)	26 (65.0)	
< 130/>175	35 (87.5)	37 (92.5)		< 3.5 / >5.5	3 (7.5)	14 (35.0)	
**RDW SD(fL)**			0.016*	**Sodium(mmol/L)**			0.37
37–50	33 (82.5)	22 (55.0)		135–145	24 (60.0)	19 (47.5)	
< 37/>50	7 (17.5)	18 (45.0)		< 135 / >145	16 (40.0)	21 (52.5)	
PLT(*10^9/L)			1.000	**cTnI(ng/mL)**			0.020*
125–350	27 (67.5)	27 (67.5)		< 0.028	15 (37.5)	5 (12.5)	
< 125/>350	13 (32.5)	13 (32.5)		> 0.028	25 (62.5)	35 (87.5)	
**CRP(mg/L)**			0.608	**Myoglobin(ug/L)**			< 0.001*
≤ 6	3 (7.6)	1 (2.5)		20.56–72.48	17 (42.5)	2 (5.0)	
> 6	37 (92.5)	39 (97.5)		< 20.56/ >72.48	23 (57.5)	38 (95.0)	
**Procalcitonin(ng/mL)**			0.074	**CK-MB(U/L)**			0.069
< 0.5	25 (62.5)	16 (40.0)		≤ 4.88	28 (70.0)	19 (47.5)	
≥ 0.5	15 (37.5)	24 (60.0)		> 4.88	12 (30.0)	21 (52.5)	
**IL-6(pg/mL)**			1.000	**PT(s)**			0.116
≤ 6.6	2 (5.0)	1 (2.5)		9.4–12.5	26 (65.0)	18 (45.0)	
> 6.6	38 (95.0)	39 (97.5)		< 9.4/ >12.5	14 (35.0)	22 (55.0)	
**ALT(U/L)**			1.000	**TT(s)**			1.000
9–50	33 (82.5)	32 (80.0)		13–21	35 (87.5)	36 (90.0)	
< 9 / >50	7 (17.5)	8 (20.0)		< 13/ >21	5 (12.5)	4 (10.0)	
**AST(U/L)**			< 0.001*	**APTT(s)**			0.516
15–40	32 (80.0)	12 (30.0)		23.5–40.7	33 (82.5)	36 (90.0)	
< 15 / >40	8 (20.0)	28 (70.0)		< 23.5/ >40.7	7 (17.5)	4 (10.0)	
**r-GTT(U/L)**			0.402	**Fibrinogen(mg/dL)**			0.802
10–60	33 (85.0)	31 (75.0)		2–5	28 (70.0)	30 (75.0)	
< 10 / >60	6 (15.0)	10 (25.0)		< 2/ >5	12 (30.0)	10 (25.0)	
**BUN(mmol/L)**			0.422	**D-Dimer(mg/L)**			1.000
3.1-8.0	11 (27.5)	7 (17.5)		≤ 0.5	0 (0.0)	0 (0.0)	
< 3.1 / >8.0	29 (72.5)	33 (82.5)		>0.5	40 (100.0)	40 (100.0)	
**HR(mean(SD))**	98.65 (19.16)	99.62 (27.07)	0.853	**SPO**_**2**_ **(mean(SD))**	94.45 (6.00)	93.12 (7.86)	0.400
**T(mean(SD))**	36.82 (0.90)	36.83 (0.69)	0.989				

(* *P* < 0.05)

**Fig. 2:**
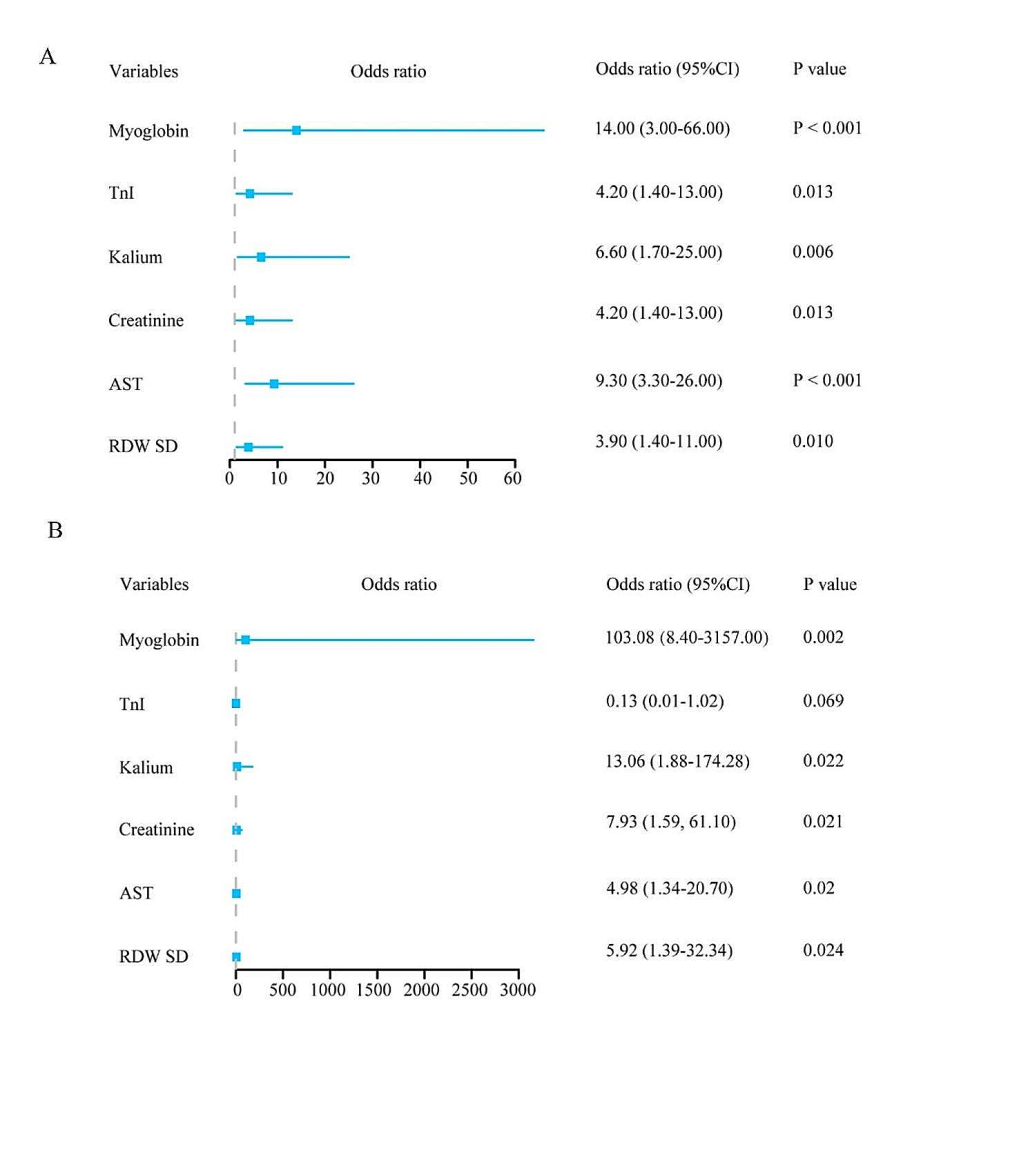
Logistic regression analysis after PSM. **A**: The outcome of univariate logistic regression analysis after PSM. **B**: The outcome of multivariate logistic regression analysis after PSM

**Fig. 3:**
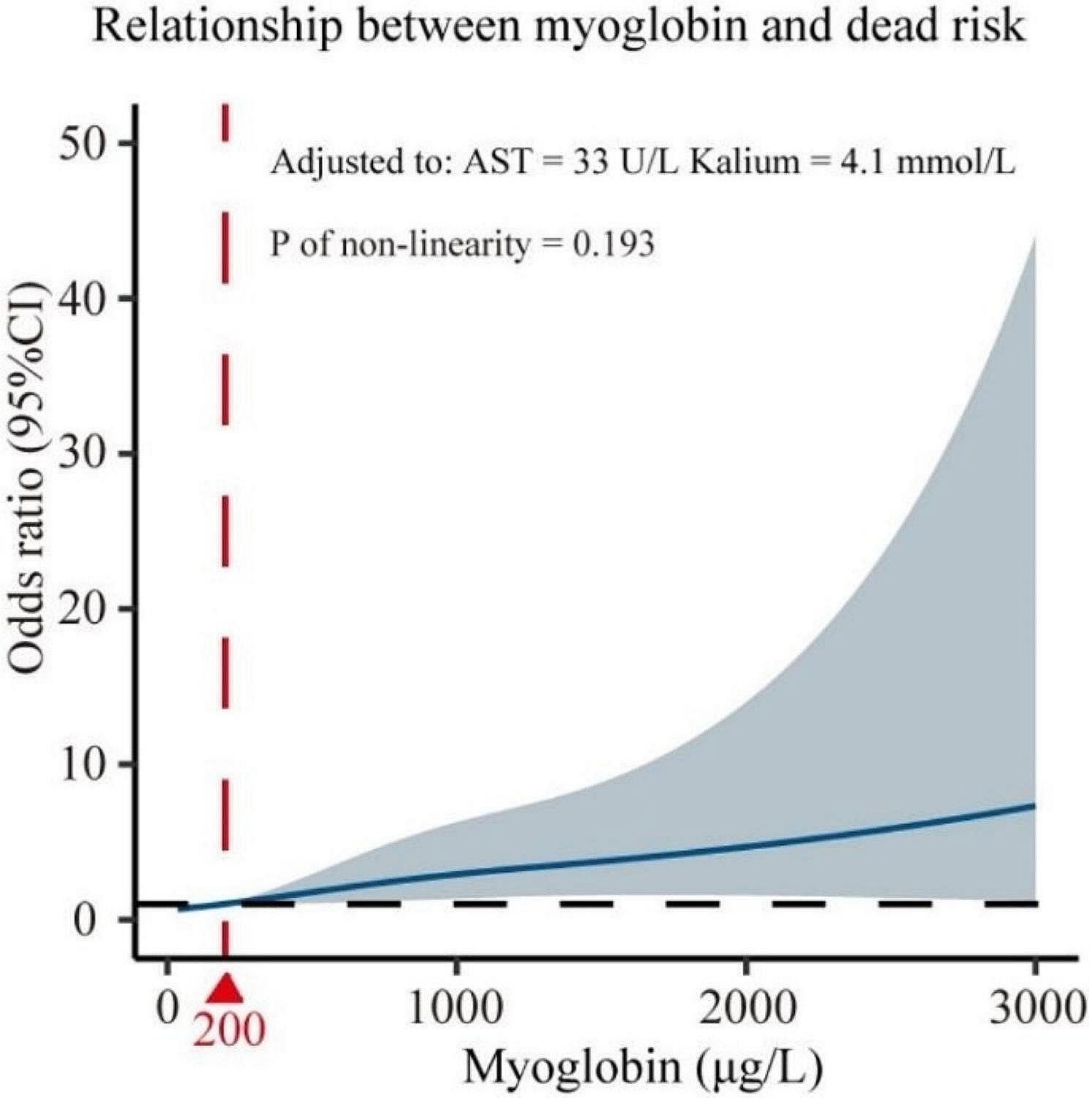
Relationship between myoglobin and dead risk

Univariate and multivariate logistic regression analyses suggested that RWD SD (OR = 5.92, *P* = 0.024), AST (OR = 4.98, *P* = 0.020), creatinine (OR = 7.93, *P* = 0.021), kalium (OR = 13.06, *P* = 0.022) and myoglobin (OR = 103.08, *P* = 0.002) were independent risk factors for death in patients (Table 6; Fig. 2). AST, creatinine and myoglobin were identified as independent risk factors for patient mortality in multivariate logistic regression analyses both before and after PSM. Therefore, after correction for AST and creatinine, we found that the relationship between myoglobin and the patient prognosis was evaluated using restricted cubic splines, and a linear relationship between myoglobin and risk of death. When myoglobin was greater than 200 ug/L, the risk of death increased in patients with critical COVID-19 infection (Fig. 3).

**Table 6:** Results of univariate and multivariate logistic regression analysis with significantly differential variants between dead and alive patients after PSM

Variants	Univariate logistic regression			Multivariate logistic regression	
	OR (95%CI)	*P* value		OR (95%CI)	Adj. *P* value
**RDW SD**	3.90 (1.40–11.00)	0.010*		5.92 (1.39–32.34)	0.024*
**AST**	9.30 (3.30–26.00)	< 0.001*		4.98 (1.34–20.70)	0.020*
**Creatinine**	4.20 (1.40–13.00)	0.013*		5.61 (1.51–23.77)	0.021*
**Kalium**	5.70 (1.90–18.00)	0.006*		13.06 (1.88-174.28)	0.022*
**cTnI**	4.20 (1.40–13.00)	0.013*		0.13 (0.01–1.02)	0.069
**Myoglobin**	14.00 (3.00–66.00)	< 0.001*		103.08 (8.40–3157.00)	0.002*

## Discussion

In this study, we retrospectively collected the data of 137 patients with critical COVID-19 infection who had been admitted to the ICU of the Fourth People’s Hospital of Shanghai, Tongji University and met the diagnostic criteria for the critical type of novel coronavirus pneumonia (trial version 10). Of the 137 patients, 84 survived and 53 died, representing a mortality rate of 38.7%, which is similar to that reported in the study by Keller et al. (38.4%, 10,391/27,053) [9]. However, this study presented a lower mortality rate than that reported by Wu et al. (60%, 15/25) [10]. The reason for the higher mortality rate in patients on ICU admission compared to patients without ICU treatment (14.8%, 21,216/149,084) [9] may be related to the increased severity of the disease.

Several independent risk factors for prognosis in patients with COVID-19 infection have been reported. However, there have been variations in their results. There were founded that age, lactate dehydrogenase, interleukin-6, and lymphocyte count were independent predictors of patient death and SPO_2_, lymphocyte count, CRP, procalcitonin, and lactate dehydrogenase levels at admission were independent risk factors for death in critically ill patients with COVID-19 infection and could be used as independent prognostic predictors to predict clinical prognosis and guide clinical management. [11–13]. In the study comprising 275 patients diagnosed with COVID-19, patients exhibiting a C-reactive protein/albumin ratio (CAR) between 1.56 and 11.19 demonstrated an eight-fold increased risk of mortality relative to those presenting with a CAR below 0.29 [14]. Furthermore, PNI has been linked to increased mortality in patients with new coronary pneumonia. A lower PNI is associated with a higher risk of death [15]. Patients with COVID-19 were prone to coagulation disorders. Disseminated intravascular coagulation (DIC) was a particularly severe form of coagulation disorder that was associated with high mortality of COVID-19 patients [16]. Previous clinical studies have indicated a correlation between D-dimer levels and disease severity in these patients. D-dimer might be a meaningful indicator of mortality [17]. Thus, as currently available studies on risk factors for death in patients with COVID-19 infection have varying results, our study focused on the risk factors for death in elder critically ill patients to help clinicians identify the risk of death at an early stage.

In this study, we revealed that abnormal AST and creatinine levels were independent risk factors for death in patients both before and after PSM. This result is consistent with the findings of researches [18, 19] which reported that AST and creatinine levels in patients with COVID-19 infection at admission were associated with disease severity and were independent risk factors for adverse outcomes. The findings [20] suggested that angiotensin-converting enzyme 2 (ACE2) is ubiquitously expressed not only in the lung and small intestine but also in the endothelial cells and smooth muscle cells of almost all organs. This further suggests that COVID-19 can mediate invasion through ACE2, leading to infection and functional damage to multiple organs. The organ dysfunction is further exacerbated by the subsequent activation of immune responses and cytokine/inflammatory storm. The hyperactivation of peripheral T cells, manifested by an increase in Th17 and high cytotoxicity of CD8 + T cells, may partially explain the severe organ dysfunction of patients [20]. In addition, the use of non-steroidal anti-inflammatory drugs, antivirals, antibiotics, and herbal medicines in improving clinical symptoms, such as fever, may also cause liver and kidney damage.

Furthermore, a positive linear relationship was detected between myoglobin levels and the risk of death in patients admitted to the ICU in this study. A previous study [21] found that myoglobin levels were lower in mildly ill patients with COVID-19 infection than in the critically ill group; however, no significant differences were identified. However, there was another research found that both gross changes in albumin and myoglobin may early disclose COVID-19 fatal outcomes [22]. Zhu et al. found that the myoglobin level must exceed the troponin level to be an independent predictor of death in patients with COVID-19 infection [23]. A clinical study by Professor Orhan Al’s team revealed that elevated troponin I levels resulting from myocardial injury were a predictor of the risk of hospitalised death in COVID-19 patients without prior cardiovascular risk factors [24]. However, 63 high-quality articles were included in a meta-analysis by Ma et al. [25]. They found that elevated myoglobin was more common than elevated cTnI in critically ill patients with COVID-19 infection. Elevated myoglobin was also an independent risk factor for severe illness [Myoglobin, OR = 13.75 (10.2–18.54) vs. cTnI, OR = 7.06 (3.94–12.65)] and death [Myoglobin, OR = 13.49 (9.30–19.58) vs. cTnI, OR = 7.75 (4.40–13.66)] in patients, compared with cTnI. Similarly, findings were reported that the serum level of myoglobin in the dead group was higher than in the survival, and an increase in myoglobin was a valuable predictor of the severity of COVID-19 [26, 27]. The results of our study are consistent with the available literature.

Myoglobin is an intracellular iron porphyrin-containing pigment protein homologous to hemoglobin found in the cytoplasm of skeletal and cardiac muscle tissues. Myoglobin stores oxygen in muscle and expels it rapidly in response to tissue hypoxia. It also participates in the process of glucose oxidation, helps myocytes transport oxygen to the mitochondria, and facilitates intracellular transport of oxygen. Myoglobin has long been considered a marker of myocardial injury; however, it is sensitive but not specific for cardiac disease [28]. Elevated myoglobin levels in critically ill patients with COVID-19 infection may increase the risk of death for the following reasons: first, when COVID-19 infection occurs, viral entry into the body’s lung tissue is mediated by ACE2 receptors, leading to alveolar oxygenation and causing hypoxia [29]. This hypoxia-induced non-specific damage to multiple organs results in increased myoglobin. Patients with critical illness have a lower oxygenation index and more severe hypoxia, and their myoglobin levels are correspondingly higher. Second, in addition to lung tissue, ACE2 is also distributed in other organs, and the virus also induces multiorgan insufficiency through ACE2 receptors in organs in the cardiovascular and renal systems [30, 31]. The inflammatory storm caused by infection will also further damage the organs, resulting in increased myoglobin. Third, when an inflammatory storm occurs in a person, myoglobin can be rapidly released into the blood in response to inflammatory stimuli [32], and the release of large amounts of myoglobin into the blood can aggravate renal function damage and, in severe cases, renal failure may occur, increasing the risk of death in patients. Fourth, De Andrade-Junior et al. [33] reported that critically ill patients with COVID-19 infection are prone to muscle atrophy and impaired muscle function, followed by elevated myoglobin levels. This study evaluated independent risk factors affecting the prognosis of critically ill patients. The results suggested that AST, creatinine, and myoglobin are independent risk factors for death in patients after eliminating the effect of confounding variables by using propensity-matched scores. In addition, the risk for death in patients with myoglobin higher than 200 ug/L was greater. This finding may help clinicians to quickly determine the possible prognosis of patients and take appropriate treatment measures in a timely manner.

In conclusion, the results of this study suggest that abnormal AST, creatinine, and myoglobin levels were independent risk factors for death in patients. This was assessed using propensity matching to eliminate the effect of underlying disease confounders. After correcting for AST and creatinine, a positive linear relationship between myoglobin and patient prognosis was detected in our analysis. Patients with critical COVID-19 infection and high myoglobin levels have a poor prognosis.

It should be noted that the study has certain limitations. Firstly, it is a single-center retrospective study with a relatively small sample size. The results need further validation. Additionally, the speed of mutation of the COVID-19 was rapid. The findings need to be further validated. Finally, the results of this study did not indicate that the use of ventilators affected the patient outcomes. This may be attributed to the relatively small sample size or the medical environment at the time of the study.

## Conclusion

High myoglobin level is an independent risk factor for death and is a prognostic marker in elder patients with severe COVID-19 infection. It may facilitate early identification of the patients who had a high risk of mortality among those presenting with critical illness, thereby enabling more rational and timely deployment of medical treatment resources.

## Data Availability

The datasets analyzed during this study are available from the corresponding author upon reasonable request.

## References

[1] The NCPE. The epidemiological characteristics of an outbreak of 2019 Novel Coronavirus diseases (COVID-19) - China, 2020. China CDC Wkly. 2020;2(8):113–22. 34594836 10.46234/ccdcw2020.032 PMC8392929

[2] Docherty AB, Harrison EM, Green CA, Hardwick HE, Pius R, Norman L, et al. Features of 20 133 Uk patients in hospital with covid-19 using the isaric who clinical characterisation protocol: prospective observational cohort study. BMJ. 2020;369:m1985. 10.1136/bmj.m1985. 32444460 10.1136/bmj.m1985 PMC7243036

[3] Wiersinga WJ, Rhodes A, Cheng AC, Peacock SJ, Prescott HC. Pathophysiology, transmission, diagnosis, and treatment of coronavirus disease 2019 (covid-19): a review. JAMA. 2020;324(8):782–93. 10.1001/jama.2020.12839. 32648899 10.1001/jama.2020.12839

[4] Yang C, Liu F, Liu W, Cao G, Liu J, Huang S, et al. Myocardial injury and risk factors for mortality in patients with covid-19 pneumonia. Int J Cardiol. 2021;326:230–6. 10.1016/j.ijcard.2020.09.048. 32979425 10.1016/j.ijcard.2020.09.048 PMC7510443

[5] Parohan M, Yaghoubi S, Seraji A, Javanbakht MH, Sarraf P, Djalali M. Risk factors for mortality in patients with coronavirus disease 2019 (covid-19) infection: a systematic review and meta-analysis of observational studies. Aging Male. 2020;23(5):1416–24. 10.1080/13685538.2020.1774748. 32508193 10.1080/13685538.2020.1774748

[6] Yang X, Yu Y, Xu J, Shu H, Xia J, Liu H, et al. Clinical course and outcomes of critically ill patients with SARS-cov-2 pneumonia in Wuhan, China: a single-centered, retrospective, observational study. Lancet Respir Med. 2020;8(5):475–81. 10.1016/S2213-2600. (20)30079-5. 32105632 10.1016/S2213-2600 PMC7102538

[7] Li X, Xu S, Yu M, Wang K, Tao Y, Zhou Y et al. Risk factors for severity and mortality in adult covid-19 inpatients in wuhan. J Allergy Clin Immunol. 2020;146(1):110-8. 10.1016/j.jaci.2020.04.006. 10.1016/j.jaci.2020.04.006 PMC7152876 32294485

[8] Wang K, Zhang Z, Yu M, Tao Y, Xie M. 15-day mortality and associated risk factors for hospitalized patients with covid-19 in Wuhan, China: an ambispective observational cohort study. Intensive Care Med. 2020;46(7):1472–4. 10.1007/s00134-020-06047-w. 32328724 10.1007/s00134-020-06047-w PMC7176814

[9] Keller K, Farmakis IT, Valerio L, Koelmel S, Wild J, Barco S, et al. Predisposing factors for admission to intensive care units of patients with covid-19 infection-results of the German nationwide inpatient sample. Front Public Health. 2023;11:1113793. 10.3389/fpubh.2023.1113793. 36875366 10.3389/fpubh.2023.1113793 PMC9975593

[10] Molins B, Figueras-Roca M, Valero O, Llorenc V, Romero-Vazquez S, Sibila O, et al. C-reactive protein isoforms as prognostic markers of covid-19 severity. Front Immunol. 2022;13:1105343. 10.3389/fimmu.2022.1105343. 36741367 10.3389/fimmu.2022.1105343 PMC9893772

[11] Donoso-Navarro E, Arribas GI, Bernabeu-Andreu FA. Il-6 and other biomarkers associated with poor prognosis in a cohort of hospitalized patients with covid-19 in madrid. Biomark Insights. 2021;16:895687437. 10.1177/11772719211013363. 10.1177/11772719211013363 PMC8150444 34103886

[12] Pan F, Yang L, Li Y, Liang B, Li L, Ye T, et al. Factors associated with death outcome in patients with severe coronavirus disease-19 (covid-19): a case-control study. Int J Med Sci. 2020;17(9):1281–92. 10.7150/ijms.46614. 32547323 10.7150/ijms.46614 PMC7294915

[13] Zhang JJ, Cao YY, Tan G, Dong X, Wang BC, Lin J, et al. Clinical, radiological, and laboratory characteristics and risk factors for severity and mortality of 289 hospitalized covid-19 patients. Allergy. 2021;76(2):533–50. 10.1111/all.14496. 32662525 10.1111/all.14496 PMC7404752

[14] Güney BÇ, Taştan YÖ, Doğantekin B, Serindağ Z, Yeniçeri M, çiçek V, et al. Predictive value of car for in-hospital mortality in patients with covid-19 pneumonia: a retrospective cohort study. Arch Med Res. 2021;52(5):554–60. 10.1016/j.arcmed.2021.02.006. 33593616 10.1016/j.arcmed.2021.02.006 PMC7874980

[15] Cinar T, Hayiroglu MI, Cicek V, Kilic S, Asal S, Yavuz S, et al. Is prognostic nutritional index a predictive marker for estimating all-cause in-hospital mortality in covid-19 patients with cardiovascular risk factors ? Heart Lung. 2021;50(2):307–12. 10.1016/j.hrtlng.2021.01.006. 33482433 10.1016/j.hrtlng.2021 PMC7832700

[16] Hayiroglu MI, Cinar T, Tekkesin AI. Fibrinogen and d-dimer variances and anticoagulation recommendations in covid-19: current literature review. Rev Assoc Med Bras. 2020;66(6):842–8. 10.1590/1806-9282.66.6.842. 32696883 10.1590/1806-9282.66.6.842

[17] Hayiroglu MI, Cicek V, Kilic S, Cinar T. Mean serum d-dimer level to predict in-hospital mortality in covid-19 patients. Eur Heart J. 2021;42:1514. 10.1093/eurheartj/ehab724.1514 34468611

[18] Bonetti G, Manelli F, Patroni A, Bettinardi A, Borrelli G, Fiordalisi G, et al. Laboratory predictors of death from coronavirus disease 2019 (covid-19) in the area of valcamonica, Italy. Clin Chem Lab Med. 2020;58(7):1100–5. 10.1515/cclm-2020-0459. 32573995 10.1515/cclm-2020-0459

[19] Siavoshi F, Safavi-Naini S, Shirzadeh BS, Azizmohammad LM, Hatamabadi H, Ommi D, et al. On-admission and dynamic trend of laboratory profiles as prognostic biomarkers in covid-19 inpatients. Sci Rep. 2023;13(1):6993. 10.1038/s41598-023-34166-z. 37117397 10.1038/s41598-023-34166-z PMC10144885

[20] Xu Z, Shi L, Wang Y, Zhang J, Huang L, Zhang C, et al. Pathological findings of covid-19 associated with acute respiratory distress syndrome. Lancet Respir Med. 2020;8(4):420–2. 10.1016/S2213-2600(20)30076-X. 32085846 10.1016/S2213-2600(20)30076-X PMC7164771

[21] de Morais BF, Puga M, Da SP, Oliveira R, Dos SP, Da SB, et al. Serum biomarkers associated with SARS-cov-2 severity. Sci Rep. 2022;12(1):15999. 10.1038/s41598-022-20062-5. 36163447 10.1038/s41598-022-20062-5 PMC9511452

[22] Ceci FM, Ferraguti G, Lucarelli M, Angeloni A, Bonci E, Petrella C, et al. Investigating biomarkers for covid-19 morbidity and mortality. Curr Top Med Chem. 2023;23(13):1196–210. 10.2174/1568026623666230222094517. 36815637 10.2174/1568026623666230222094517

[23] Zhu F, Li W, Lin Q, Xu M, Du J, Li H. Myoglobin and troponin as prognostic factors in patients with covid-19 pneumonia. Med Clin (Engl Ed). 2021;157(4):164–71. 10.1016/j.medcle.2021.01.014. 34458579 10.1016/j.medcle.2021.01.014 PMC8378829

[24] Cinar T, Hayiroglu MI, Cicek V, Kilic S, Asal S, Dogan S, et al. Prognostic significance of cardiac troponin level in covid-19 patients without known cardiovascular risk factors. Am J Emerg Med. 2021;45:595–7. 10.1016/j.ajem.2020.12.033. 33349488 10.1016/j.ajem.2020.12.033 PMC7833992

[25] Ma C, Tu D, Gu J, Xu Q, Hou P, Wu H, et al. The predictive value of myoglobin for covid-19-related adverse outcomes: a systematic review and meta-analysis. Front Cardiovasc Med. 2021;8:757799. 10.3389/fcvm.2021.757799. 34869669 10.3389/fcvm.2021.757799 PMC8636904

[26] Luo J, Xie C, Fan D. Is it meaningful for serum myoglobin in patients with covid-19 decreased? Georgian Med News. 2023(338):102–3. 37419480

[27] Lazar M, Barbu EC, Chitu CE, Anghel AM, Niculae CM, Manea ED, et al. Mortality predictors in severe SARS-cov-2 infection. Med (Kaunas). 2022;58(7). 10.3390/medicina58070945. 10.3390/medicina58070945 PMC9324408 35888664

[28] Kurt-Mangold M, Drees D, Krasowski MD. Extremely high myoglobin plasma concentrations producing hook effect in a critically ill patient. Clin Chim Acta. 2012;414:179–81. 10.1016/j.cca.2012.08.024. 22960204 10.1016/j.cca.2012.08.024

[29] South AM, Diz DI, Chappell MC. Covid-19, ace2, and the cardiovascular consequences. Am J Physiol Heart Circ Physiol. 2020;318(5):H1084-90. 10.1152/ajpheart.00217. 2020. 10.1152/ajpheart.00217.2020 PMC7191628 32228252

[30] He Q, Mok TN, Yun L, He C, Li J, Pan J. Single-cell rna sequencing analysis of human kidney reveals the presence of ace2 receptor: a potential pathway of covid-19 infection. Mol Genet Genomic Med. 2020;8(10):e1442. 10.1002/mgg3.1442. 32744436 10.1002/mgg3.1442 PMC7435545

[31] Han X, Xia N, Chen Z, Pan C, Huang X. Inpatients with brain damage, impaired airways, and severely restricted daily activities have an increased infection rate during the covid-19 pandemic: a single-center retrospective analysis from Wuhan. Am J Phys Med Rehabil. 2020;99(10):884–6. 10.1097/PHM.0000000000001535. 32744819 10.1097/PHM.0000000000001535 PMC7406202

[32] Hendgen-Cotta UB, Kelm M, Rassaf T. Myoglobin functions in the heart. Free Radic Biol Med. 2014;73:252–9. 10.1016/j.freeradbiomed.2014.05.005. 24859377 10.1016/j.freeradbiomed.2014.05.005

[33] de Andrade-Junior MC, de Salles I, de Brito C, Pastore-Junior L, Righetti RF, Yamaguti WP. Skeletal muscle wasting and function impairment in intensive care patients with severe covid-19. Front Physiol. 2021;12:640973. 10.3389/fphys.2021.640973. 33776796 10.3389/fphys.2021.640973 PMC7991788

